# Correspondence

**Published:** 1872-08

**Authors:** 


### CORRESPONDENCE.

*Ed. Dental Register:*

Last week it was decided that I must undergo a severe, but not tedious surgical operation. This, to a man already in bed, was not very pleasant, but the decision had been given by a veteran of more than fifty years' experience, and so I prepared for the execution of the sentence.

I was aided, next morning, from the bed to the carriage, proceeded to my office, took nitrous oxyd, had the operation performed, and returned without serious demoralization.

And now, a few words about this: A large and deep crucial incision was to be made through a tumor (a carbuncle) on the neck. Seated on the footstool of an operating chair, my head supported, I inhaled the gas to complete anæsthesia, *told* the surgeon when to cut, knew as well when he cut as if nothing had been taken, walked to an adjacent sofa at the close, and suffered no pain whatever. At no time was there any mental or physical commotion from the effects of the gas. No rapidity of thought, no ringing of the ears, no quickening of the pulse, no feeling of suffocation, or even want of breath, no disposition to move, no dread of the operation, no lack of confidence in the anæsthetic power of the gas, and, I am told there was no change in complexion or countenance.

Nor is this all: Ever since that fearful ordeal of 1865, with which you are familiar, I labor under a state of chronic hyperæsthesia, and with me, this had to be overcome, in addition to what the gas would have had to do for an ordinary patient. It is impossible to accurately estimate this additional power required. That it is considerable, may be inferred from the fact that my feet suffer as severely from walking on rough surfaces, with boots on, as they formerly did when walking barefoot.

Now all this conveys to you no new idea. You are aware that just as decided experiments were made at my rooms on Seventh street, Cincinnati; but many of our profession are so incredulous that they must have "line upon line." Nor is it anything that may not *ordinarily* be obtained in the use of nitrous oxyd, when administered by judicious hands.

Some, however, will claim that this was only a favorable, if not an accidental case. It is unpleasant to tell some things; but the demands of science override personal whims. You are aware that I have always suffered from defective respiration, and may also know that, in consequence of this, there is recently dilatation of the right side of the heart; you know also that I am plethoric, and your readers know that I am not young. What, then, is left to make mine a favorable case?

But will our profession, or the medical, ever learn that anæsthesia and unconsciousness are totally different things? I fear not. I saw two of your (and my) best friends, eminent physicians in your city, give a man chloroform, not fit to use except as a local application. He became wildly delirious, but was held by assistants until the operation was completed. They appeared perfectly satisfied with the result, as the patient did not remember anything, from which I infer it was nothing unusual in their experience, which was quite extensive. I was much discouraged by the slow progress made by anæsthetic science.

Sometime, however, it will be generally recognized that anæsthesia should not be entrusted to charlatans, and that however much a man may know on other subjects, he will remain a fool in regard to this, until he gives it proper study.

In a recent case that excited a good deal of interest, it appears the patient was dosed with *her own breath* and something else, called nitrous oxyd. A sow submitted to such administration would have a right to complain of worse treatment than was given to her progenitors, by the devils, on the shores of Galilee, for who would not prefer to be drowned in the clear waters of Genesarat, rather than be smotherd in her own excrements? G. W.

Xenia, Ohio, June 7, 1872.

